# Ethnobotanical study of wild edible plants in Burji District, Segan Area Zone of Southern Nations, Nationalities and Peoples Region (SNNPR), Ethiopia

**DOI:** 10.1186/s13002-016-0103-1

**Published:** 2016-08-02

**Authors:** Mersha Ashagre, Zemede Asfaw, Ensermu Kelbessa

**Affiliations:** 1 Department of Biology, Faculty of Computational and Natural Sciences, Bule Hora University, P.O. Box 144, Bule Hora, Ethiopia; 2 Department of Plant Biology and Biodiversity Management, Addis Ababa University, PO Box 1176, Addis Ababa, Ethiopia

**Keywords:** Burji District, Biodiversity, Ethnobotany, Indigenous knowledge, Wild edible plants

## Abstract

**Background:**

An ethnobotanical study of wild edible plants was conducted in Burji District, Segan Area Zone of Southern Nations, Nationalities and Peoples Region, Ethiopia. The objective of the study was to identify and document wild edible plants and the associated ethnobotanical knowledge of the local people.

**Methods:**

Relevant ethnobotanical data focused on wild edible plants were collected using guided field walk, semi-structured interview, and direct field observation. Informant consensus method and group discussion were conducted for crosschecking and verification of the information. Both descriptive statistics and quantitative ethnobotanical methods were used for data analysis.

**Results:**

We documented 46 species distributed in 37 genera and 29 families based on local claims of use as food. Local users collect most of these plants from the wild. The common plant families that encompass more number of wild edible plant species were Anacardiaceae (five species) followed by Boraginaceae, Fabaceae and Solanaceae which contributed three species each.

**Conclusion:**

The study showed the existence of a number of wild edible plants which mitigate food insecurity situations during problematic times that the people of the area face occasionally. Informants stated that wild growing edible plants are under threat due to increased anthropogenic pressure and disturbed climatic conditions. This calls for urgent and collaborative actions to keep the balance between edible plants availability in the wild and their utilization by the community. Furthermore, the study attempted to prioritize very important wild edible plants as perceived by the local people for possible domestication and/or sustainable utilization.

## Background

The value of wild edible plants to sustain people in different parts of the world has been well documented [22, 43]. Though many more wild edible plant species are believed to be undocumented to date, 413 species of wild/semi-wild species used by the people in Ethiopia have been recorded [32]. Edible fruit bearing species form one of the most important local survival strategies. This is particularly important because their consumption has been reported to be more common and widespread in food insecure areas [21, 23]. Although agricultural societies mainly depend on conventional crop plants, the tradition of eating wild edible plants has not been completely abandoned and their nutritional roles and health benefits are being reported in many studies worldwide [9, 31]. It is disputed that past societies made more use of the wild flora to overcome hunger than is done today [16, 30]. In spite of their importance, wild edible plants, especially fruit bearing species, suffer notable disregard from research and development plans in Ethiopia, particularly in Burji District of Southern Nations, Nationalities and Peoples Region. Thus, they remain inadequately documented in the study area.

Basic information pertaining to wild fruit species is available from the local people who are the custodians of these resources and knowledge about them [48]. At present, due to the catastrophic destruction of their natural habitats, wild edible plant resources are degrading fast along with the associated indigenous knowledge. Assessment and better understanding of the wild food resources and associated knowledge is crucial. As a step in this direction, the study makes use of local peoples’ knowledge to define the cultural domain of wild fruits and other edibles. Cultural domains are the key starting points for studying peoples’ perceptions of the natural world; and are important aspects of local indigenous knowledge by which cultural settings are understood (Puri and Vogl, 2005 (A methods manual for ethnobotanical research and cultural domain analyisis with analysis using ANTHROPC (Unpublished)). Hence, defining cultural domains from an emic perspective enables us to elicit lists of cultural domain elements that are considered by the local people as being members of a particular domain [11, 12]. Elements of a cultural domain can be understood through free-listing method [33], which has been successfully used by several researchers for eliciting cultural domains or as a precursor for further studies [4, 13, 37, 41].

Since long time ago, wild edible plants are an integral part of local culture and support the food requirement of different communities. Besides its deep cultural rooting, one reason for this is the problem of food security. Their important role that these plants can play to reduce poverty through improving household food security and incomes has been recognized [19, 45]. Different ethnobotanical studies in Africa revealed that wild edible plants are essential components of many African diets especially in periods of seasonal food shortage [5, 9, 17, 47]. A study conducted in Zimbabwe revealed that some poor households rely on wild fruits as an alternative to cultivated food for a quarter of all dry season’s meals [53].

On the other hand, the knowledge of the community is currently eroded and lost due to loss of traditional cultural systems and conversion of rangelands and forest ecosystems to other land use types. The role of wild edible plants in developing countries has been ignored and under-estimated for many years [23, 49]. For example, a study conducted in southern Ethiopia by [23] and in Afar Region by [20] indicated that strong traditions, beliefs, and religious taboos still limit people’s psychological and mental willingness to domesticate and cultivate wild food plants. As a result, the indigenous knowledge, practice, and skill associated with wild edible plants is highly developed, but it is poorly investigated and documented [21, 35]. This indigenous knowledge, practice and skill is gradually being eroded and lost due to urbanization, industrialization as well as mobility of youth from rural settings [21]. The contribution that many wild edible plants make to many poor people’s livelihoods is not commonly acknowledged in many national statistical reporting [39]. This clearly indicates the absence of knowledge and interest in policy makers about wild resources. But these neglected groups of food plants can contribute their part in poverty reduction, ensuring food security, increasing agricultural diversification and income generation and this study aims the case that in addition to cultivated crops, better attention should be given to wild edible plants.

Thus, the development, promotion, and wider utilization of wild edible plants particularly in the dry land areas undoubtedly resolve the food insecurity problems. To achieve this objective, wild edible plants could be integrated to the dry land agro forestry system and home gardens to improve people’s livelihoods and maximize their income sources as well. Various studies suggested a need for urgent documentation of indigenous knowledge related to plants use as wild food to make it available to future generations. Thus, intensive ethnobotanical research plays a vital role to draw information on plants and related indigenous knowledge for conservation and sustainable utilization. Since the use of wild edibles varies across cultures, more studies are required. There are few or no documents on indigenous knowledge and practice with wild and semi-wild edible plant species in the remote parts of southern Ethiopia where their use is very prominent both at times of excess and food deficiency [1]. Likewise, there is no such wild edible plants research and documentation carried out in Burji District, Segan Area Zone of Southern Nations, Nationalities, and Peoples Region. In this study therefore, wild edible plants and indigenous knowledge of the Burji people on use and management of these plants in fulfilling food requirements and the existing threats to the plants were documented. This is believed to add up to the country’s database of wild edible plants and in documenting indigenous knowledge of the people.

## Methods

### Study area

**Fig. 1:**
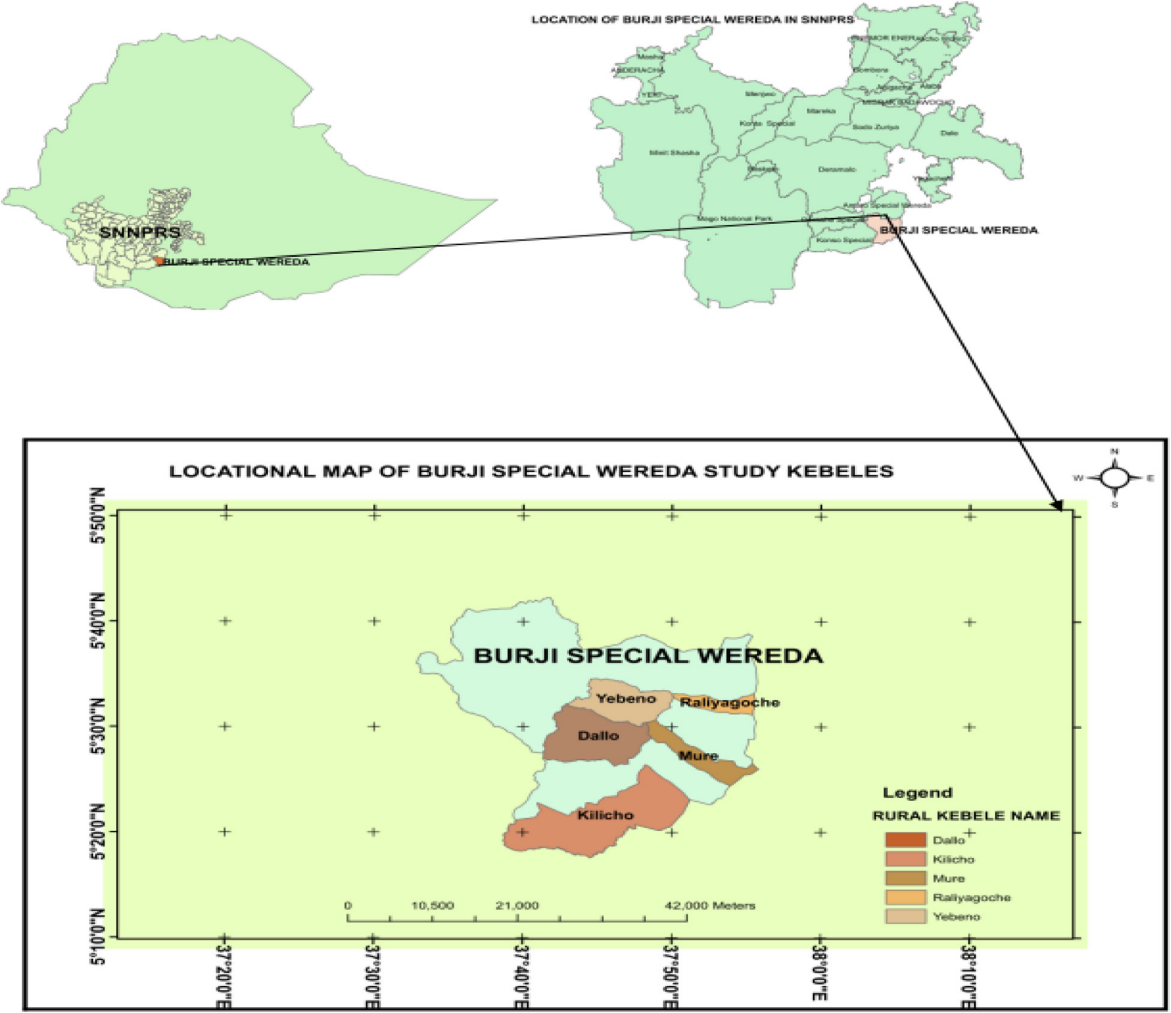
Map of Ethiopia showing Burji District (the study area)

This study was conducted in Burji District which is located 530 km away from Addis Ababa to the South and 260 km away from Hawasa, the capital of Southern Nations, Nationalities, and Peoples Region (SNNPR). Burji is one of the districts in Segan Area Zone of the SNNP Region of Ethiopia which is named after the Burji people, who have their homeland in this district. It has two town kebeles (the smallest administrative units) and twenty four rural kebeles (farmers’ associations) with an area of 1,128.40 km^2^. This district is bordered by Oromia Region to the east and south, Konso District to the west and Amaro District to the north (Fig. 1).

### Sampling design and informant selection

A reconnaissance survey was made from October 15 to 30, 2013 and five kebeles (study sites) based on the difference of their altitude (lowland, midland and highland) were selected purposively. Data collection trip was made starting from November 25, 2013 and wild edible plants reported as food by the informants were collected.

A total of 45 informants between the age of 12 and 85 were used for this study [33] indicated that when recording indigenous knowledge held by certain social groups the choice of key informant is crucial. Accordingly, eight key informants were selected using purposive sampling technique to pick those individuals who are knowledgeable about wild edibles at least one key informant from each kebele to get the required important information about wild edible plants. The purposive sampling technique is a type of non-probability sampling that is most effective when one needs to study a certain cultural domain with knowledgeable experts within. The key informants include knowledgeable persons from all age groups and were selected with the help of local dwellers, society leaders, and developmental agents. The remaining 37 informants were selected randomly from five kebeles, seven to eight informants from each kebele to known how much wild edible plants knowledge is disseminated among the community members. Following this, ethnobotanical data were collected, following [14, 15, 33]. Semi-structured interviews, guided field walk, discussions, market surveys, and field observation, with randomly picked and key informants were applied based on a checklist of questions. The selected informants in the sample site were interviewed using semi-structured interview focusing on the wild edible plants, their management and uses such as how they came to know these plants can be eaten; How they manage negative side effects on users; Which plant is more preferable in its test and use? Have you got any economic benefit from wild edibles? What do you suggest about the current conservation status of these plants? What are the contributions of wild edible plants in fulfilling food shortage or missed nutrients in the diet? Which groups of the community commonly collect and use wild edibles (adult & old men, women or children)? etc. Semi-structured interview questionnaires were prepared and conducted after translating into the local (Burji) language. It was an important tool for the collection of both qualitative and quantitative data at the same time. The informants participated in answering the questions including by showing the plants that they used as food during guided field walk interview.

A brief group discussion was made with the informants at each kebele and site focusing on the status of the vegetation and acceptance of wild edible plants by the community. Full notes on facts and information about the respondents, history of wild food collectors, history of wild edible plants, and other essential information (based on the questionnaire) were recorded on site. During the discussion the informants were free to state about wild edible plants and their knowledge without being interfered.

### Plant specimen collection and identification

For ethical reasons, ethnobotanical data were collected in the presence of local administrators and with the permission of each informant for the publication of the research. Good specimens (those bearing flowers and/or fruits) of all the wild edible plants identified by the informants were collected as voucher specimens. Collection was made with the informants during guided field walk. Along with collection, the field activities included taking notes on the plants and the associated indigenous knowledge with preliminary identification of the family and sometimes the species when possible. The necessary information about the plants such as habit, habitat, altitude, latitude and longitude and features that is specific to each plant. Each specimen was given a collection number and scientific and/or local name when possible. Information was also captured with photographs to document the sites, individual plants, the edible parts, and actions of users showing how they manage them. Standard procedure was followed in pressing the specimens, which were then brought to the National Herbarium (ETH), Addis Ababa University where they were allowed to dry, deep-frozen and determinations made using taxonomic keys and descriptions given in the relevant volumes of the Flora of Ethiopia and Eritrea such as [18, 25]. Further refining of determinations was made by visual comparison with authenticated herbarium specimens and finally checking the accuracy by a senior plant taxonomist. The plant specimens with labels were finally deposited at the ETH and the resulting data of the study presented in tables, graphs, and percentages.

### Data analysis

Both qualitative and quantitative analytical tools were used for data analysis following the approaches of [33]. Microsoft Excel spread - sheet was employed for organizing some ethnobotanical data. Preference ranking was performed to analyze most popular and preferred wild edible plants, at least in the context of the people who used them during food shortage in the area. Peoples’ preferences of wild edible plants were undertaken with informants to determine their order of cultural importance across a community. Likewise, direct matrix ranking was used to order wild edible plants by considering their several attributes one at a time. After identifying seven wild edible plants based on their high use values as perceived by ten informants, paired comparison was employed as described by [33].

Informant consensus was used to identify the plants most cited by the informants and this method of prioritizing the wild edible plants was used to evaluate the reliability of the data. Furthermore, informant consensus factor (ICF) was used to find out the most common wild edible plants in the district following the approach of [26] using the following formula:

$$ ICF=\frac{n_{ur}-{n}_t}{n_{ur}-1} $$

Where, n_ur_ - number of use-reports in each category and n_t -_ the number of taxa used. The product of this factor ranges from 0 to 1. A high value (close to 1) indicates that relatively few taxa (usually species) are used by a large proportion of people, while a low value indicates that the informants disagree on the taxa to be used for different purposes commonly. Fidelity level (FL) was used to quantify the importance of a species for a given purpose using the following formula:

$$ \mathrm{F}\mathrm{L}=\mathrm{I}\mathrm{p}/\mathrm{I}\mathrm{u}\;\mathrm{and}\;\mathrm{F}\mathrm{L}\%=\left(\mathrm{I}\mathrm{p}/\mathrm{I}\mathrm{u}\right)\times 100 $$

where, Ip = the use of a species for the same major purpose; Iu = the total number of informants who mentioned the plant for any use. Comparison of wild edible plant knowledge in gender wise as well as among different social groups in the community was also computed.

## Results

### Basic information about diversity of wild edible plants in the study area

**Fig. 2:**
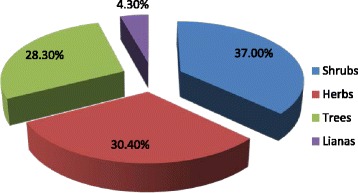
Growth habits of wild edible plants

The presence of some remnant plant species indicates that the study area is rich in plant biodiversity. However, the environment is highly affected due to extensive agricultural activities and high density of population within a small district. The area yielded a total of 46 wild edible plant species belonging to 37 genera and 29 families. Among them, 17 (37 %) were shrubs, 14 (30.4 %) were herbs, 13 (28.3 %) were trees and three (4.3 %) were lianas (Fig. 2). This adds up to about 72 % wild edible woody plant species. The family Anacardiaceae had the highest proportion of wild edible species represented by five species and Boraginaceae, Fabaceae and Solanaceae contributed three species each. These plants, which were collected in the altitudinal range of 1481 – 2325 m, a. s. l. serve different purposes to the communities. The difference in species diversity was mainly due to differences in altitude, which in turn depends on the soil, temperature, and rainfall, that are determining factors for the survival and growth of species.

### Parts of wild edible plants used and mode of preparation as food

Widely used plant parts of wild edibles by the local people in the study area include fruits, leaves, roots, and stems. Maximum numbers of species were harvested for their fruit, followed by leaves and roots and other parts cover less percentage (Table 1). Most of the plant parts (40, 87 %) were eaten uncooked (raw) while some of them (3, 6.5 %) needed processing and cooking to make them suitable for consumption and few of them (3, 6.5 %) could be eaten either cooked or uncooked.

**Table 1:** Plant parts used as food

Parts used	No. of wild edible plants	Percent
Fruits	33	71.6
Leaves	5	10.9
Roots	4	8.7
Bark	1	2.2
Nectar	1	2.2
Seed	1	2.2
Stem	1	2.2

### Informant consensuses on most frequently used wild edible plants in the study area

Some wild edible plants were well known in the study area more than others. As a result, local informants cited the most commonly used plants repeatedly as supplementary foods to the staple food. For example, *Arisaema schimperianum* Schott was cited by 43 of the informants as a supplementary item to the staple food and *Rhus tenuinervis* Engl. by 31 in the same way. These and other most widely used wild edible plants are listed and described in Table 2.

**Table 2:** Informant consensus on most commonly used wild edible plants

Scientific name of wild edible plants	No. of informants	Percentage
*Arisaema schimperianum*	43	93.5
*Rhus tenuinervis*	31	67.4
*Ficus sur*	25	54.4
*Carissa spinarum*	23	50.0
*Syzygium guineense* subsp*. guineense*	16	34.8
*Ximenia americana*	15	32.6
*Eriosema schirene*	14	30.4
*Cordia africana*	12	26.1
*Balanites aegyptiaca*	10	21.7
*Eriosema nutans*	8	17.4

### Informant consensus factor (ICF)

Informant consensus means agreement among informants. Selecting wild edible plants by using informant consensus was used to evaluate the reliability of the data. When the ICF of the above selected wild edibles was calculated, the number of use citations in each species (n_ur_) was 28, and the number of selected species used (n_t_) was 10. Hence, ICF becomes 0.7. This product is close to one and it indicates that relatively few species are used for different purposes by a large proportion of the local community.

### Preferences for some wild edible plants

Preference ranking was conducted to rank some selected wild edible plants based on the degree of their importance in using them at different times. Following the methods of [33], each informant was asked to think; order and rank the items based on their personal preference, community importance, or any other criteria set by him/her and this helps to indicate the most preferred wild edible plant by the community as a food. Thus, ranking of five wild edible plants which were used at different times (Table 3) made by ten informants showed that *Arisaema schimperianum* with a total score of 46 out of 50 possible points ranked first and hence was the most preferred wild edible plant at any time. Since the knowledge on the use of wild edibles differ from person to person, the output of the comparison showed that the informants perceived the plants differently in many cases as it emerged from the scores they gave.

**Table 3:** Preference ranking of five selected wild edible plants based on their use as perceived by informants

		Informants (R_1_ - R_10_)										
Wild edible plant species	R_1_	R_2_	R_3_	R_4_	R_5_	R_6_	R_7_	R_8_	R_9_	R_10_	Total	Rank
*Arisaema schimperianum*	4	5	5	4	4	5	5	4	5	5	46	1^st^
*Carissa spinarum*	3	4	3	5	4	3	5	4	5	3	39	2^nd^
*Cordia africana*	1	4	1	2	3	1	2	2	1	2	19	5^th^
*Ficus sur*	2	1	3	2	3	4	3	5	3	4	30	3^rd^
*Rhus tenuinervis*	1	3	2	1	4	3	1	2	3	2	22	4^th^

### Pair wise comparison of wild edible plants

Pair wise comparison was used to evaluate the degree of preference or levels of importance of seven selected wild edible plants. Ten informants (six key and four randomly taken informants) ranked seven wild edible plants (Table 4) and the results showed that *Physalis peruviana* L., *Rubus steudneri* Schweinf, *Opuntia ficus - indica* (L.) Miller, *Flacourtia indica* (Burm.f.) Merr and *Lantana viburnoides* (Forssk).Vahl. stood 1^st^, 2^nd^, 3^rd^, 4^th^ and 5^th^ respectively. *Pappea capensis* Eckl. & Zeyh and *Myrsine africana* L. were less preferred and less used compared to the rest five species. Pair wise ranking can be used for evaluating the degree of preference or level of importance of selected plants or plant parts.

**Table 4:** Pair wise ranking of seven wild edible plants

	Informants (R_1_ - R_10_)											
Wild edible plant species	R_1_	R_2_	R_3_	R_4_	R_5_	R_6_	R_7_	R_8_	R_9_	R_10_	T	R
*Flacourtia indica*	4	1	4	5	3	4	6	6	5	0	38	4^th^
*Lantana viburnoides*	1	3	5	4	3	2	0	5	3	2	28	5^th^
*Myrsine africana*	2	6	2	0	2	4	2	0	2	1	21	7^th^
*Opuntia ficus - indica*	0	6	4	6	6	4	5	6	4	1	42	3^rd^
*Pappea capensis*	6	2	1	4	2	1	2	6	1	2	27	6^th^
*Physalis peruviana*	6	1	6	6	6	5	6	1	6	5	48	1^st^
*Rubus steudneri*	4	5	6	4	1	4	6	5	4	6	45	2^nd^

### Use diversities of wild edible plants collected from the study area

All 46 wild edible plants documented in the study area were reported to have additional uses other than their use as food. The additional use categories included; fodder with 35 species, fuel with 28 species, construction with 11 species, medicine with nine species, utensils with six species and live fence with two species according to their importance. The complete list of uses of all wild edible plants collected from the study area is given in Table 5.

**Table 5:** List of wild edible plants used by the local people of the study area and their uses other than being edible

Family	Scientific name (Genus/species)	Local name	Gf	Latitude	Longitude	Alt	Ae	Ab	Pu	Adu	Vou.No.
Acanthaceae	*Acanthus eminens* C.B.Clarke	Hoxoxa	Sh	37 N0368056	UTM0609995	2318	Hl	R	Ne	Fw	MA07
Amaranthaceae	*Amaranthus caudatus* L.	Raso	H	37 N0371700	UTM0605786	1926	Ml	R	S	Fo	MA26
Anacardiaceae	*Lannea schimperi* (A. Rich.) Engl.	Anderaku	T	37 N0376345	UTM0612789	1718	Ml	R	Fr	Fw,Fo	MA31
	*Rhus ruspolli* Engl.	Dodobay	Sh	37 N0368035	UTM0609990	2313	Hl	R	Fr	Co,Fw	MA11
	*Rhus tenuinervis* Engl.	Qadhadhiya	Sh	37 N0378004	UTM0612513	1642	Ml	R	Fr	Co,Fw	MA46
	*Rhus vulgaris* Meikle	Qadhadhiya	Sh	37 N0368035	UTM0609990	2313	Hl	R	Fr	Co,Fw	MA09
	*Sclerocarya birrea* (A.Rich) Hochst	Boita	T	37 N0377913	UTM0612224	1693	Ml	R	Fr	Fw,Fo	MA39
Apocyanaceae	*Carissa spinarum* L.	Agama	Li	37 N0366187	UTM0604511	2006	Ml	C	Fr	Fw,Fo	MA18
Araceae	*Amorphophallus gomboczianus* ^a^										
	Pichi.Serm.	Laye	H	37 N0366904	UTM0604919	2052	Ml	R	Rt	Fo	MA15
	*Arisaema schimperianum* Schott	Hidha	H	37 N0368026	UTM0609800	2293	Ml	R	Rt	Fo,Co	MA14
Balanitaceae	*Balanites aegyptica* (L.) Del.	Angalda	T	37 N0377647	UTM0612652	1668	Ml	C	Fr	Fw,Co	MA29
Boraginaceae	*Cordia africana* Lam.	Mearera	T	37 N0368421	UTM0610194	2322	Hl	C	Fr	Co,Fw	MA05
	*Cordia ellenbeckii* Gurke	Dela’a	Sh	37 N0366907	UTM0594820	1481	Ll	R	Fr	Fw,Fo	MA42
	*Ehretia cymosa* Thonn	Kolisha	T	37 N0377912	UTM0612585	1653	Ml	R	Fr	Fw,Fo	MA27
Brassicaceae	*Brassica rapa* L.	Tenjilo	H	37 N0378004	UTM0612513	1661	Ml	C	Fr	Fo	MA36
	*Raphanus raphanistrum* L.	Bedhaka	H	37 N0378004	UTM0612513	1661	Ml	R	Le	Fo	MA37
Cactaceae	*Opuntia ficus - indica* (L.) Miller	Gambora	Sh	37 N0378027	UTM0612510	1670	Ml	R	Fr	Lf	MA35
Ebenaceae	*Euclea divinorum* Hiern	Measka	Sh	37 N0377203	UTM0612726	1679	Ml	R	Fr	Co,Fw	MA38
Euphorbiaceae	*Bridelia scleroneura* Muell.Arg.	BuneGalday	Sh	37 N0366960	UTM0595328	1538	Ml	R	Fr	Fo,Fw	MA44
	*Flueggea virosa* (Wild.) Voigt	Qarchechelo	Sh	37 N0377994	UTM0612641	1661	Ml	R	Fr	Fw,Fo	MA28
Fabaceae	*Acacia hockii* De Wild	Lanqey	Sh	37 N0366045	UTM0604602	1970	Ml	R	Ba	Fo	MA21
	*Eriosema nutans* Schinz	Kurte	H	37 N0367162	UTM0594992	1494	Ll	R	Rt	Fo	MA40
	*Eriosema schirene* Bak.f.	Qamura	H	37 N0366787	UTM0604817	2050	Ml	R	Rt	Fo,Co	MA16
Flacourtiaceae	*Dovyalis abyssinica* (A.Rich.) Warb.	Longo	Sh	37 N0368026	UTM0609800	2293	Ml	R	Fr	Fw,Fo	MA13
	*Flacourtia indica* (Burm.f.) Merr	Dunadunise	T	37 N0377004	UTM0612734	1713	Ml	R	Fr	Co,Fw	MA34
Lamiaceae	*Fuerstia africana* T.C. E.Fr.	Sinaqayish	H	37 N0366660	UTM0605031	2070	Ml	C	Le	Fo	MA22
	*Satureja abyssinica* (Benth.) Briq.	Shusha	H	37 N0366721	UTM0604847	2056	Ml	C	Le	Fo	MA17
Moraceae	*Ficus palmata* Forssk.	Guma	T	37 N0368035	UTM0609990	2313	Hl	C	Fr	Fo,Fw	MA10
	*Ficus sur* Forssk.	Elisho	T	37 N0368497	UTM0610151	2317	Hl	R	Fr	Fw,Fo	MA04
Myricaceae	*Myrica salicifolia* A. Rich	Buddule	T	37 N0376751	UTM0612762	1690	Ml	R	St	Co,Fw	MA30
Myrsinaceaae	*Myrsine africana* L.	Chuchurina	Sh	37 N0368042	UTM0610074	2294	Ml	C	Fr	Fw,Fo	MA06
Myrtaceae	*Syzygium guineense subsp.*										
	*guineense* (Wild.) Dc.	Shelelay	T	37 N0366131	UTM0604558	1990	Ml	R	Fr	Co,Fw	MA20
Olacaceae	*Ximenia americana* L.	Mullancho	Sh	37 N0366149	UTM0604536	1928	Ml	R	Fr	Fw,Fo	MA19
	*Ximenia caffra* Sond	Ingigita	Sh	37 N0367209	UTM0594956	1495	Ll	R	Fr	Fw,Fo	MA41
Oleaceae	*Olea europea subsp. cuspidata*										
	(Wall.ex G.Don) Cif.,L’Oliv.Coltore	Yeger	T	37 N0376345	UTM0612789	1718	Ml	R	Fr	Co,Fw	MA32
Polygonaceae	*Rumex abyssinicus* Jacq.	Hopho	H	37 N0368541	UTM0610195	2303	Hl	R	St	Fo	MA02
Rosaceae	*Rubus steudneri* Schweinf	Shaqar	Li	37 N0368516	UTM0610191	2305	Hl	R	Fr	Fo	MA03
Rubiaceae	*Canthium lactescens* Hiern	Bolocket	Sh	37 N0367217	UTM0595709	1559	Ml	R	Fr	Fw,Fo	MA45
Santalaceae	*Osyris quadripartita* Decn.	Tunqa	Sh	37 N0368051	UTM0609994	2325	Hl	R	Fr	Fw,Md	MA08
Sapindaceae	*Pappea capensis* Eckl. & Zeyh	Biqa	T	37 N0370869	UTM0605753	1993	Ml	R	Fr	Co,Fw	MA24
Solanaceae	*Physalis peruviana* L*.*	Tunaye	H	37 N0368432	UTM0610185	2315	Hl	C	Fr	Fo	MA01
	*Solanum nigrum* L.	Tunaye	H	37 N0369716	UTM0605792	2076	Ml	C	Fr	Fo	MA23
	*Solanum villosum* L.	Tunaye	H	37 N0371700	UTM0605786	1926	Ml	C	Le	Fo	MA25
Sterculiaceae	*Sterculia africana* (Lour.) Fiori	Qereri	T	37 N0366617	UTM0594882	1495	Ll	R	Fr	Fw,Fo	MA43
Thymelaeaceae	*Gnidia somalensis* (Franch.) Gilg.	Anbura	H	37 N0376345	UTM0612789	1718	Ml	C	Rt	Fo,Md	MA33
Verbanaceae	*Lantana viburnoides* (Forssk).Vahl.	Qarqando	Sh	37 N0368048	UTM0609991	2323	Hl	R	Fr	Fo	MA12

**Key**: **Growth form** (Gf), *T* Tree, *Sh* Shrub, *Li* Liana, *H* Herb; **Parts used** (Pu), *Le* Leaf, *Rt* Root, *St* Stem, *Ba* Bark, *Fr* Fruit, *S* Seed, *Ne* Nectars; **Abundace** (Ab), *C* Common, *R* Rare; **Agro** - **ecology** (Ae), *Hl* Highland, *Ml* Mid-land, *Ll* = Lowland; **Additional uses** (Adu), *C* = Construction, *Fw* Firewood, *Fo* Forage, *Md* Medicinal, *Ut* Utensils, *Lf* Live fence; **Voucher Number** (Vou. No.); (^a^ = Endemic)

### Direct matrix ranking

Direct matrix ranking was made based on the use diversities of wild edible plants which were selected by the key informants. Hence, six multipurpose plant species were taken out of the total wild edible plants and eight use categories were considered for the assessment of their relative importance or uses in the informants’ respective localities. The eight use values include medicinal, forage, food, firewood, construction, charcoal, fencing, and furniture making. Each informant was asked to assign use values (5 = best, 4 = very good, 3 = good, 2 = less used, 1 = least used and 0 = not used). The use values of the six multipurpose wild edible plant species were recorded, average use value for the species taken and the scores of each species summed up and ranked. The results of this analysis are given in Table 6.

**Table 6:** Average score for direct matrix ranking of six wild edible plant species based on their general use values (5 = best, 4 = very good, 3 = good, 2 = less used, 1 = least used and 0 = not used

		Use categories								
Plant species	Md	For	Fd	Fw	Co	Ch	Fe	Fur	T	R
*Balanites aegyptiaca*	4	4	3	4	3	4	5	3	30	4^th^
*Cordia africana*	4	4	5	5	5	3	4	5	35	1^st^
*Ehretia cymosa*	4	3	2	4	3	1	3	4	24	5^th^
*Euclea divinorum*	3	1	2	4	2	1	3	0	16	6^th^
*Olea europaea* subsp*. cuspidata*	5	3	4	5	4	3	4	3	31	3^rd^
*Syzygium guineense* subsp *guineense*	4	4	5	5	5	4	4	2	33	2^nd^
Total	24	19	21	27	22	16	23	17	169	
Rank	2^nd^	6^th^	5^th^	1^st^	4^th^	8^th^	3^rd^	7^th^		

This investigation showed that, *Cordia africana* Lam., *Syzygium guineense* subsp*. guineense* (Wild.) Dc. and *Olea europaea* subsp*. cuspidata* (Wall.ex G.Don) Cif.,L’Oliv.Coltore were ranked 1^st^, 2^nd^ and 3^rd^ and hence were the most preferred wild edible plants by the local people for various uses and were the most threatened species as the informants reported, which was evidently seen by their scarce distribution and time required to collect voucher specimens of these species. *Balanites aegyptica* (L.) Del. and *Ehretia cymosa* Thonn were ranked 4^th^ and 5th respectively. On the other hand, *Euclea divinorum* Hiern was the least ranked species as a multipurpose plant and was the less threatened and a species of no concern in the area.

The values for use reports across the selected species were summed up and ranked. The results showed that the local people harvested multipurpose species mainly for firewood, medicine, fencing, construction, food, and forage with the rank of 1^st^, 2^nd^, 3^rd^, 4^th^, 5^th^ and 6^th^ respectively. Thus, sustainable use of these top-ranked species is under questions, as the pressure on their consumption was intensified, superimposed on lack of propagation techniques in the area. This was evidenced by the high rate of loss of *Syzygium guineense* subsp*. guineense* in the area. Generally, the use matrix ranking showed that these wild edible plants were at conservation risk because of overexploitation for their additional uses for different needs.

### Fidelity level index of food potential of wild edible plants

Fidelity level (FL) quantifies the importance of a species for a given purpose. Hence, fidelity level values were calculated for the following most commonly used individual wild edible plants at different times to fulfill food requirements: *Arisaema schimperianum*, *Rhus tenuinervis*, *Ficus sur* Forssk., *Carissa spinarum* L. and *Syzygium guineense* subsp*. guineense*. These wild edible plants had the highest FL values which could be an indication of their potential value as food (Table 7). *Arisaema schimperianum* had the highest fidelity level, which indicated its importance in supplementing food requirements of the local community and needs due consideration about its conservation and bringing it into cultivation with certain scientific investigation on its improvement.

**Table 7:** The relative food potential value of five wild edible plants

No.	Wild edible plant	Time in which the food is used	Ip	Iu	FL	FL%	Ra
1	*Arisaema schimperianum*	To supplement the staple food	43	43	1	100	1^st^
2	*Rhus tenuinervis*	To fill the gap	28	31	0.9	90	4^th^
3	*Ficus sur*	To fill the gap	23	25	0.92	92	3^rd^
4	*Carissa spinarum*	To fill the gap	22	23	0.96	96	2^nd^
5	*Syzygium guineense*	To fill the gap	16	18	0.89	89	5^th^

### Comparison of wild edible plants knowledge among different social groups in the community

Wild edible plant knowledge among different social groups may not be the same and this is confirmed after doing different analyses. Mostly, women and children usually go out into the field and forests to collect a variety of leaves, roots, seeds, and fruits. The analysis made by taking five individuals from the three age groups (10 - 30, 31 - 51 and >51) starting from the bottom of the informant list to the top showed that there were significant differences among average numbers of wild edible plants cited by youngsters and elders. This provides us with information that as the age increases wild edible plants knowledge also increases in relative terms. In the same way differences of wild edible plant knowledge with respect to education level of the informants was analyzed by taking the first ten uneducated and ten educated informants from the list. The results of the analysis depicted the presence of traditional wild edible plant knowledge difference among literate and illiterate members of the informants. This again provides us with information necessary to predict wild edible plant knowledge with respect to education level (being educated increases traditional wild edible plant knowledge). In addition, differences of wild edible plant knowledge with respect to sex difference were computed. This analysis was done by taking the first ten females and ten males from the list of informants randomly and it confirmed that females were more knowledgeable in traditional wild edible plant practice than males. Hence, it was observed that age, level of education and gender were factors that have influenced knowledge on the use of wild edible plants. Higher averages were calculated for women than men, for older people than younger, for literate people than illiterate ones. When we compare the number of wild edible plants cited by eight key and eight randomly taken informants who cited relatively highest number of wild edible plants those mentioned by key informants were much greater (about 87) than those cited by randomly taken informants (about 57).

### Habitats and abundance of wild edible plants in the study area

In this study, wild edible plants were collected from various habitats including roadsides, live fence, crop fields, grazing land, forests, woodland and riverside and the proportions are shown in Table 8. The majority (84.8 %) of wild edible plants that the communities reported were collected from the wild habitats. They were growing mostly in disturbed habitats, mainly in woodlands and grazing lands. Since most wild edibles were found in the wild, a big threat was seen to their existence with the current rate of habitat destruction and conversion. This in turn resulted in rarity of some wild edible plants such as *Arisaema schimperianum*, *Cordia ellenbeckii* Gurke, *Sclerocarya birrea* (A.Rich) Hochst, *Sterculia africana* (Lour.) Fiori, *Syzygium guineense* subsp. *guineense*, *Ximenia americana* L. and *X. caffra* Sond (Table 8). They were becoming highly scarce in the study area, because they were sought for forage, construction, fuel and other uses in the locality. Hence, such pressure calls for urgent measures to be taken to rehabilitate and conserve the remaining vegetation in general and wild edible plants in particular.

**Table 8:** Distribution of wild edible plants in different habitat

Habitat type	No. of wild edible plants	Percent	Degree of management
Wood land	13	28.3	Uncultivated
Grazing land	11	23.9	Uncultivated
Roadside	8	17.4	Uncultivated
Forest	6	13.0	Uncultivated
Crop fields	4	8.7	Semi - cultivated
Live fence	3	6.5	Semi - cultivated
Riverside	1	2.2	Uncultivated
Total	46	100.0	

## Discussion

**Fig. 3:**
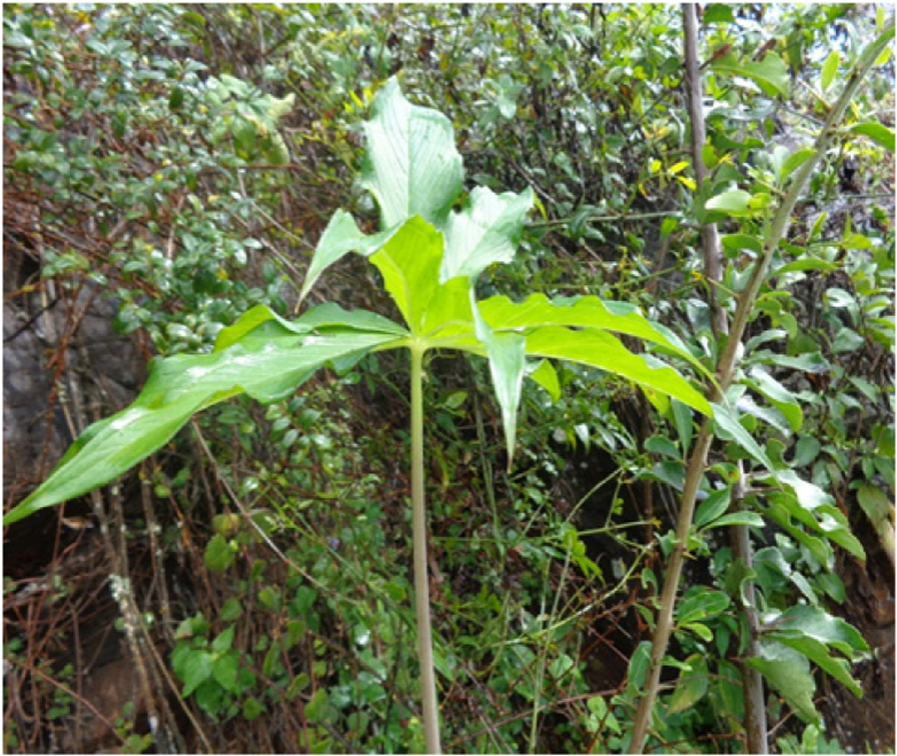
*Arisaema schimperianum* (*Hidha*) (Photograph taken from the study area)

Relatively high number of wild edible plants was documented from the study area. The total number of species, 46, of wild edible plants reportedly consumed in this study is lower than some of those reported from other studies within Ethiopia such as [1] reported 137 wild edible species used by the Konso ethnic community in Southern Ethiopia and [9] who documented 66 edible plant species in Derashe and Kucha Districts in Southern Ethiopia. The result, however, is comparable closely with that of [44] who reported 58 wild edible species used by the Oromo ethnic community in Chelia District, West - Central Ethiopia. The possible explanation for these differences could be the differences of local traditions and customs of using these plants. Anacardiaceae had the highest proportion of wild edible species represented by five species and Boraginaceae, Fabaceae and Solanaceae contributed three species each. The present study has shown that wild edible plants are an integral part of the diet of local people of the study area at times of both food plenty and scarcity. The findings of this study revealed that wild edible plants were collected from a variety of habitats such as woodlands, grasslands, roadsides, forests, and fallow lands. Similar results were reported by [28, 44, 55] who said that in Chelia District, West - Central Ethiopia and Eastern Usambara of Tanzania wild food plants were collected by village communities from forests, bushlands, secondary forests, and fallow lands. The result of this study made known that the most common harvested growth forms of wild edible plants were shrubs and herbs. This could be due to their presence in high diversity in the district. The majority of wild edible plants (76 %) have reproductive parts (nectars, fruits and/or seeds) as edible parts, while only 24 % of them are vegetative parts (leaves, stems, barks and roots) reported as edible parts. This report is in agreement with earlier study by [6, 8] who reported that 72 % and 82 % of species have reproductive parts as edible parts respectively. Most of the edible plant parts (87 %), were eaten raw without any further processing (cooking and spicing) by the local communities. Similar trends were observed in other areas of the country by [2, 28] in Alamata, Cheha, Goma and Yilmana Denssa Districts; by [9] in Derashe and Kucha Districts and by [54] around Dheeraa town. Fruits and leaves were the most reported plant parts consumed by the local people. Most fruits are eaten raw as snacks such as between meals while herding livestock or collecting firewood. Similar results were reported by [2, 3, 38, 42, 51] as fruits and leaves had higher preference by the respective communities. It is reported that wild edible plants commonly used during hungry periods of the day, seasonally during periods of scarcity or extreme famine, or to moisten the mouth in the absence of drinking water and to add variety of the diet. The extent of wild edible plants utilization varied with respect to age, sex, and season. The findings of this study revealed that these plants were usually collected by children and women which are similar reports to [10, 24, 44, 52]. The results of this study also showed that women on average knew and reported more wild edible plants than their male counterparts. This finding is also in line with the reports of [40, 44, 46]. This investigation indicated that the knowledge of wild edible plants increased with the age of the respondents which entail that the younger generations have to some extent little knowledge of wild edible plants than the elderly people. This could be due to the low interest of the younger generation to know more about wild edible plants or due to less exposure to the wild environment since youngsters now a day spent more of their time at schools. This finding is in agreement with the reports of [29, 44]. This result is in contrast to the reports of [34, 54] in which younger generations were more knowledgeable of wild edible plants around Dheeraa Town, Arsi and in Northern Ethiopia. A significant difference of wild edible knowledge was observed between key and randomly taken informants (key informants were more knowledgeable than the others). In the same way literates were more knowledgeable than illiterate informants, findings in line with the reports of [44]. But this result is contrasted with the finding reported by [34] where education is not the important factor responsible for variation of knowledge of wild edible plants. When we consider some of the wild edible plants, especially those used both in cooked and uncooked form as in *Arisaema schimperianum* (*Hidha* in Burji language), begin to sprout/emerge from the soil around mid March which indicates the start of the rainy season and its root comes to maturity up to the end of May. Informant from the culture and tourism office of Burji District said, its preparation needs great care because if its stem juice (jelly - like fluid) touch the skin (especially between the fingers), tongue or throat, it burns forming rash like that of scabies and upon scratching it would be changed to wound. But, this wound can be cured by treating it with salt solution. The roots are collected, washed and chopped with knife and ground with stone mill and allowed to ferment after it has been wrapped with *Ensete ventricosum* (Welw.) Cheesman leaves. The fermented dough could be baked as bread (*Kussa*) having very attractive flavor and aroma or could be prepared as porridge or cultural foods such as (*Fiqe*). On the other hand, its dough could be mixed with grain powder to make a cultural drink called (*Biirqaa*) without cooking it. Key informants said, its usage was mainly during the dry or (*Bonii)* season and previously it was one of the stable food items and not food of problematic time and was very preferable food item culturally in Burji ethnic group. It could be stored from June - October without being damaged if properly wrapped and tied with dried *Ensete ventricosum* leaves. It is commonly reproduced from the tuber through cutting. Elder key informants confirm that this plant was plenty when farmers used to use hand digging system because they were not uprooted. But when they started the use of oxen farming the plant’s survival ability decreased since it was being uprooted by the plough (Fig. 3).

**Fig. 4:**
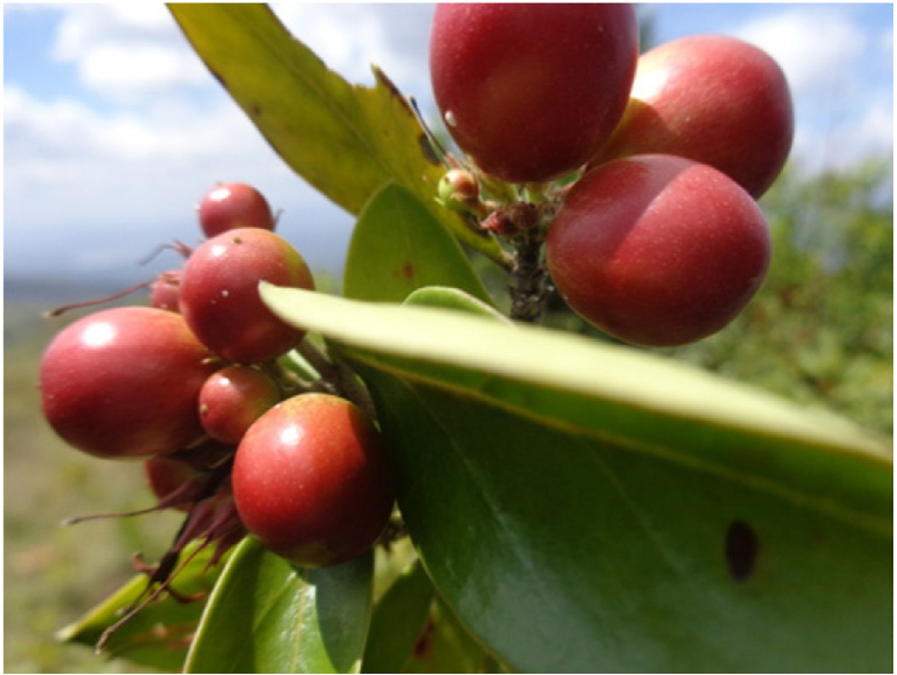
*Carissa spinarum* (*Agama*)

**Fig. 5:**
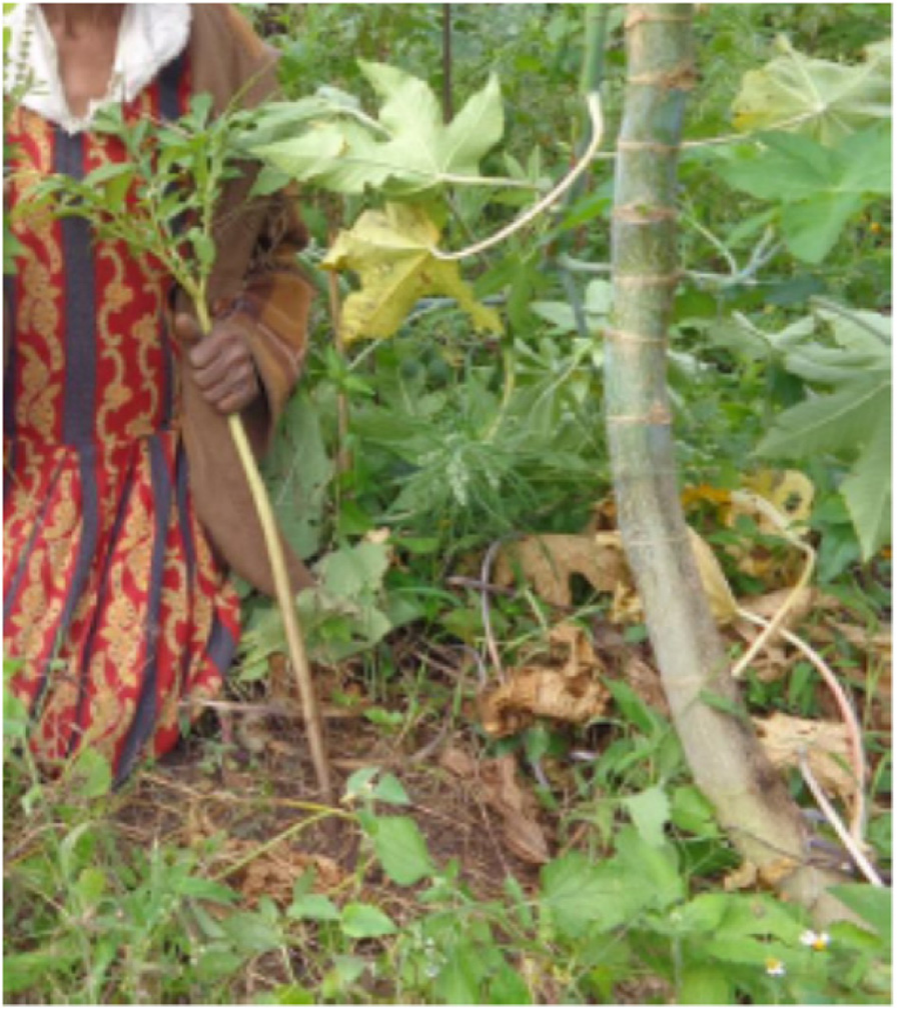
*Amorphophallus gomboczianus* (*Laye)*

Informant and field guide from Dallo Kebele said, the green fresh fruits of *Carissa spinarum* (*Agama* in Burji language) were collected, washed and boiled until dissolved. After filtering, it was used to make porridge and eaten by adding salt. On the other hand, black fresh ripe fruits were eaten without cooking (Fig. 4 - unripe fruit). They also said that the leaves of *Solanum villosum* L. (*Tunaye* in Burji language) were cooked and eaten like those of other vegetables while its fresh ripe blue -black fruits are eaten raw. A powdered seed of *Amaranthus caudatus* L. (*Raso* in Burji language) was among those plants eaten after cooking as pan cake (*Budenii*), porridge and used for local beer called (*Birqa*) sometime by mixing it with some grain flour. The root of *Amorphophallus gomboczianus* Pichi - Serm (*Laye* in Burji language) (Fig. 5) was washed, chopped, dried and powdered to mix it with other grain flour to make porridge, pan cake and cultural foods like (*Kurkufa*) and (*Fiqe*). It burns the hand when processing it in its fresh (wet) form but not as such harmful. The leaves of *Raphanus raphanistrum* L. (*Badhaka* in Burji language) were cooked like that of cabbage and eaten with pan cake, bread or other cultural foods. The fruits of *Myrsine africana* L. (*Chuchurina* in Burji language) were also used as food and medicine to expel ascaris in children.

One of the elder informants said that people used to chew the leaves of *Rhus tenuinervis* (*Qadhadhiya* in Burji language) many years ago before *Catha edulis* (Vahl) Forssk. ex Endl. was introduced into Burji District, especially when they planned to travel long distances. On the other hand, its fresh ripe yellow fruits were eaten. Mature, dried fruits of *Sterculia africana* (*Qarari* in Burji language) having nearly similar taste with groundnut (*Arachis hypogaea* L. - commonly called *Ocholoni* in many local languages). The fruits are delicious when eaten and are used commonly even if they are becoming rare at present. Since the stem of this plant is smooth and difficult to climb, people prefer it for beehive - hanging to protect the honey from different attackers. Women and children have more exposure, experience, and responsibility than men in the management, harvesting, processing, and sale of edible wild plants. Similar report was made by [49] and this may be due to a number of factors including occupation, culture, place of work, interaction existing between individuals which influence plant experience and knowledge both in age and gender among individuals [14].

### Traditional management practices and threats to wild edible plants

Since the Burji people are highly agrarian and produce different types of crops especially *Eragrostis tef* (Zucc) Trotter, *Zea mays* L. and *Phaseolus vulgaris* L. to supply dwellers of the surrounding districts, they expand their farming land by clearing the woodland forests, which is ongoing to date. So, agricultural expansion, overgrazing and fuelwood collection were found to be the most threatening factors. Ethnobotanical investigations done in Ethiopia [1, 9, 27] also reported similar pattern of threat factors to wild edible plants and associated traditional knowledge. The output of a direct matrix ranking exercise showed highest values/ranks for a number of multipurpose wild edible plants of the study area including *Cordia africana, Syzygium guineense* subsp*. guineense* and *Olea europaea* subsp*. cuspidata*. The result indicated that these plants were exploited more for their non-food uses than for reported food values. Overharvesting of multipurpose wild edible plant species for fuel wood, medicine, fencing, construction, and forage purposes were found the responsible factors aggravating depletion of the species in the area. Thus, the result calls for an urgent complementary conservation action to save the fast eroding multipurpose wild edible plant species of the area [1] also reported the same pattern of highest exploitation of wild edible plants for uses other than their food values in south Ethiopia. In fact, there is a trend to leave some wild edible plants such as *Flacourtia indica* and *Rubus steudneri* species as a shade and live fence when they clear forests for agricultural activities. Even though the topography of the district is rugged, the farmers use different traditional soil and water conservation mechanisms such as terracing, horizontal ploughing, digging drainage ditches, and live fencing. They also protect different plant species in the communal land and in spiritual areas such as in the compounds of churches and mosques. There are certain restrictions on the use of some plant species which is controlled by the elders of this ethnic group traditionally. For instance, the cutting of big trees, mainly wild edible and medicinal trees and shrubs for firewood, is forbidden. On the other hand, the keeping of individual beehive-hanging trees, which pass from father to son, is a common tradition and management practice of this ethnic group. Nowadays, soil and water conservation activities are taking place as one of the rural development programmes in the district even if not as strong as in other places. According to the respondents and as it is mentioned above, the expansion of farming land is the major threat, followed by overgrazing. This result is consistent with the finding of [23] and [54] in which the challenges facing wild edible plants have been reported. Hence, strategies should be designed to protect and domesticate these plants for future uses.

## Conclusion

Most of the identified wild edibles have been reported to be edible in other parts of Ethiopia. In an ethnobotanical study in some selected districts of Ethiopia such as [2] recorded 20 of the wild edible plant species which were reported in this study, while a study undertaken in three districts of Amhara Region [34], indicated the use of 16 of the wild edible species which were mentioned here. This shows that many of the plants are popular as edibles even beyond the present study area. In this study one endemic species (*Amorphophallus gomboczianus)* and nine additional species (*Acanthus eminens* C.B.Clarke*, Brassica rapa* L.*, Canthium lactescens* Hiern*, Cordia ellenbeckii*, *Eriosema nutans* Schinz*, Eriosema schirene* Bak.f.*, Gnidia somalensis* (Franch.) Gilg.*, Lantana viburnoides* and *Solanum villosum)* are recorded which were not mentioned in the list of 413 wild edible plants reported by [32].

The transfer of local knowledge within the community on wild edible plants is not differentiated by gender or age and enables knowledge continuity. It is believed that traditional wild food represents the identity of a certain ethnic group and wild food plants could be used during periods of ample food production to supplement the staple food or to fill the gap of seasonal food shortage as well as during famine. Ethiopia’s ambitions to create healthy and productive environments, and communities enjoying food security as well as food sovereignty could be realized through effective application of indigenous knowledge and practices. In addition to their food value, some species of wild edible plants have other economic values. This study showed that, of the 46 identified wild edible species *Arisaema schimperianum* and *Amorphophallus gomboczianus* (4.4 %) were used to supplement the regular food supply, 40 species (87 %) were used to fill the gap of seasonal food shortage and seven species (*Dovyalis abyssinica* (A.Rich.) Warb., *Ehretia cymosa*, *Euclea divinorum*, *Ficus sur* Forssk., *Lannea schimperi* (A. Rich.) Engl., *Olea europaea* subsp*. cuspidata* and *Rumex abyssinicus* Jacq. (15.2 %) were recorded in the category of plants consumed during famine. According to [1], the number of species and plant parts used for food by all age and gender groups increases at times of famine or food shortage resulting from domestic conflicts. Famine foods are used only when preferred alternatives are not available, and in situations where chronic food shortage prevails [9, 21, 23]. The preferred and commonly used wild edible plants were however becoming rare due to population pressure. This has been exacerbated by the increasing incidence of climate change especially by human induced factors.

In addition to their use for household consumption, some wild edible plants such as *Arisaema schimperianum*, *Syzygium guineense* subsp*. guineense,* and *Ximenia americana* were truly observed being sold in the local market of the study area to support household incomes. This result is in agreement with earlier studies by [9] in Derashe and Kucha Districts, by [7] in Benna-Tsemay District and by [27] in semiarid east Shewa Zone. The marketability of wild edible plants in the study area was very low due to small production and supply which is also reported by [27]. This is because, mature roots of *Arisaema schimperianum* could be available from June to October; ripe fruits of *Syzygium guineense* subsp*. guineense* could be found from March to May while that of *Ximenia americana* could be available from April to June and November to October. According to [9], income derived from the sale of wild plant is of particular importance to the poorer households, which must supplement food production with cash in order to meet their basic needs. The density and production potential of wild edible plants in the study area was very small due to the people being highly agrarian and their extensive use of land for farming activities (to date woodland clearing continues in the lowland of Segan area). According to [20] agricultural expansion affects resource availability in rural areas thereby decreasing the volume of fruit harvestable for private consumption and sale and what is seen in the study area is in agreement with this fact. The low price and inadequate market supply of wild and semi-wild fruit species discouraged traders from marketing this resource and also hampered the promotion of trade.

The dominance of fruits as edible parts (71.7 % - Table 9) found in the present study has also been reported in most previous studies undertaken in Ethiopia such as [2, 6, 9, 50]. On the other hand, [36] reported that leaves and stems are the most widely used parts of wild edible trees and shrubs in the West Bank of Palestine. This difference might be due to variation in the available species, and culture of the communities with respect to food preference and preparation. As regards the mode of consumption, 40 species were consumed raw or without further processing, three were consumed cooked or roasted and three species were consumed either raw or cooked. This result is in agreement with the findings of previous studies conducted in Southern Ethiopia [9, 23]. Most of the wild edible trees and shrubs that require further processing are consumed as emergency food at times of chronic food shortages.

**Table 9:** List of families, genera and species with their edible parts (Le = Leaf, Rt = root, St = Stem, Ba = Bark, Fr = Fruit, S = Seed, N = Nectars)

Family name	No. of genera	No. of species	Percent	Parts used as food						
				N	Le	Rt	St	Ba	Fr	S
Acanthaceae	1	1	2.2	*						
Amaranthaceae	1	1	2.2							*
Anacardiaceae	2	5	10.9		*				*	
Apocynaceae	1	1	2.2						*	
Araceae	2	2	4.4			*				
Balanitaceae	1	1	2.2						*	
Boraginaceae	2	3	6.5						*	
Brassicaceae	2	2	4.4		*					
Cactaceae	1	1	2.2						*	
Ebenaceae	1	1	2.2						*	
Euphorbiaceae	2	2	4.4						*	
Fabaceae	2	3	6.5			*		*		
Flacourtiaceae	2	2	4.4						*	
Lamiaceae	2	2	4.4		*					
Moraceae	1	2	4.4						*	
Myricaceae	1	1	2.2				*			
Myrsinaceae	1	1	2.2						*	
Myrtaceae	1	1	2.2						*	
Olacaceae	1	2	4.4						*	
Oleaceae	1	1	2.2						*	
Polygonaceae	1	1	2.2				*			
Rosaceae	1	1	2.2						*	
Rubiaceae	1	1	2.2						*	
Santalaceae	1	1	2.2						*	
Sapindaceae	1	1	2.2						*	
Solanaceae	2	3	6.5		*				*	
Sterculaceae	1	1	2.2						*	
Thymeliaceae	1	1	2.2			*				
Verbanaceae	1	1	2.2						*	

## Recommendations

Ethnobotanical studies are important to promote the conservation and management of the vegetation of a certain area. The loss of indigenous knowledge on wild edible plants may occur if the resources disappear from the landscape. Being a basic source of information about the types of wild edible plants found in the study area and their use, this study would help in maintaining the ecological balance of the area and serve as a wakeup call for other researchers, including ethnobotanists and ecologists, to proceed to more of such studies. It enriches the herbarium and serves as permanent herbarium records and specimens for determination and quick botanical reference in future. In addition to these:

- Some plants, for example, *Ariseama schimperianum* could be a very good food source at any time; hence should be given due attention either in maintaining it or improving it through domestication for more intensive usage.
- Proper consideration should be given in the conservation and keeping of both wild edible plants and associated indigenous knowledge.
- Expansion of farm lands through clearing forests and woodlands should be stopped by inducing intensive agricultural activities than extensive one through fulfilling different inputs.
- The local people need awareness raising interventions about the sustainable use of natural resources.
